# Applying the CORE20PLUS5 to Address Health Inequalities for Patients Under the Rehabilitation and Recovery Service in the London Borough of Hackney

**DOI:** 10.1192/bjo.2024.548

**Published:** 2024-08-01

**Authors:** Ailbhe Brennan

**Affiliations:** East London NHS Foundation Trust, London, United Kingdom

## Abstract

**Aims:**

The NHS England Core20PLUS5 aims to reduce national healthcare inequalities by identifying five clinical areas requiring accelerated improvement for the most deprived 20% of the population. Three of these clinical areas are: Severe Mental Illness (SMI), Early Cancer Diagnosis and Maternity Care.

Hackney has the highest proportion of areas within the most deprived 10% nationally. The Hackney Rehabilitation and Recovery Team is a specialist service for those with SMI. While the service does not provide maternity care it is uniquely placed for women's health outreach work in this population. Research has shown that lower participation by those with SMI in screening may make them 2.5 times more likely to die prematurely from cancer. Bearing this in mind, this project aimed to improve early cancer diagnosis and management of women's health to improve health inequalities for females with SMI in Hackney.

**Methods:**

I audited cancer screening compliance from the medical records of the 19 female patients under the Hackney Rehabilitation and Recovery Team and obtained patient feedback to explore barriers to access screening. I used a pool of possible keywords to perform a search for any discussion of women's health issues during contact with mental health professionals. Encouraging a culture of ‘Making Every Contact Count’, I presented the results of this audit at a Team Education Session, after which attendees received a personalised list detailing their caseload's outstanding health needs as identified from the audit. I led a weekly physical health clinic which addressed women's health issues. I designed a referral pathway for patients with complex psychiatric needs with the local cervical screening service which allows for longer appointments.

**Results:**

16% of the female patients under the care of the Hackney Rehabilitation and Recovery Service had never had a discussion covering women's health issues. 73% of mammograms and 53% of smear tests were outstanding. Barriers to access include a lack of knowledge of cancer screening programmes and practical issues in booking appointments. Some cited a lack of confidence in travelling to appointments and communication issues (access to a mobile phone, email address or post) as an issue.

**Conclusion:**

Designing interventions to boost the uptake of cancer screening appointments for female patients with SMI is a practical application of the CORE20PLUS5 approach. An MDT approach including patient participation and feedback is key when developing effective outreach initiatives.

